# Lights Out in the Canopy: Arboreal Camera Traps Offer New Insights Into Sleeping‐Site Partitioning and Sleep Ecology in Primate Communities

**DOI:** 10.1002/ece3.74525

**Published:** 2026-10-28

**Authors:** Olivier Kaisin, Mathéo Pochez, Cédric Vermeulen, Laurence Culot

**Affiliations:** ^1^ Laboratório de Primatologia, Departamento de Biodiversidade, Instituto de Biociências Universidade Estadual Paulista (UNESP) Rio Claro São Paulo Brazil; ^2^ Gembloux Agro‐Bio Tech, Forest Is Life – TERRA Teaching and Research Center University of Liège (ULiège) Gembloux Belgium

**Keywords:** arboreal camera trapping, Atlantic Forest, coexistence, sleep ecology, sleeping‐site selection, spatiotemporal overlap

## Abstract

Sleep is an essential yet costly biological process, leaving animals vulnerable to predation and constraining their use of space and time. Optimal sleeping sites therefore represent valuable, potentially contested resources, yet they remain largely overlooked in primate community ecology. Understanding how syntopic species compete for and partition sleeping sites can reveal mechanisms of coexistence, but community‐level studies remain limited because multiple arboreal species are difficult to monitor simultaneously. Here, we used arboreal camera traps to investigate sleeping‐site use and spatiotemporal partitioning within a Brazilian Atlantic Forest primate community. Over 7 months (2024–2025), we monitored ten *Copaifera trapezifolia* trees used as preferred sleeping sites by southern muriquis ( *Brachyteles arachnoides* ), brown howler monkeys ( *Alouatta guariba* ) and black‐tufted capuchins (*Sapajus nigritus cucullatus*), recording 4195 videos documenting 1253 visits and 158 sleeping nights. *C. trapezifolia* trees constituted a shared but limited resource, and access reflected predicted interspecific differences in resource holding potential (RHP), the relative ability of a species to prevail in direct contests over a shared resource. Muriquis and capuchins, the higher‐RHP species with larger home ranges, showed high temporal overlap in copaíba use—preferentially sleeping there on warmer nights and under lower lunar illumination, suggesting roles for thermoregulation and perceived predation risk—offset by reduced spatial overlap, consistent with greater ranging flexibility enabling spatial avoidance. Howlers, constrained by more restricted ranging and repeatedly displaced in direct encounters with the other species, instead relied on temporal segregation to access the same preferred trees. Nocturnal videos further revealed activity beyond residual wakefulness at the edges of the night, offering rare direct evidence of nocturnal behaviour and fragmented sleep in wild arboreal primates. These findings indicate that sleeping sites can function as contested, high‐value resources structuring primate community dynamics through asymmetric contest competition, and highlight arboreal camera trapping as a powerful tool for studying primate sleep ecology.

## Graphical abstract

Using arboreal camera traps, we show that sleeping sites represent a shared but contested resource in a Brazilian Atlantic Forest primate community, and that spatiotemporal resource partitioning is driven by resource holding potential (RHP). Although syntopic primates relied on the same *Copaifera trapezifolia* trees as key sleeping sites, access reflected predicted differences in RHP: higher‐ranked species achieved high temporal overlap by spatially avoiding one another, while lower‐ranked species, repeatedly displaced in direct encounters, relied on temporal segregation to access the same preferred trees. Nocturnal recordings further provided rare evidence of fragmented sleep in wild arboreal primates, highlighting the ecological importance of sleeping sites as contested resources and the value of canopy camera trapping for studying primate sleep ecology.

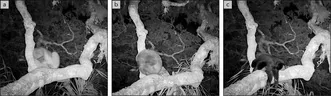

## 1 Introduction

Understanding the mechanisms enabling species coexistence remains a central challenge in ecology (McPeek 2025). Tropical ecosystems are particularly well suited to address this question, harbouring a disproportionately large share of terrestrial biodiversity (Myers et al. 2000). Within these systems, non‐human primates (hereafter primates) contribute substantially to vertebrate biomass and frequently occur in diverse multi‐species assemblages (Culot et al. 2019; Kamilar and Beaudrot 2013). Primates provide valuable models for investigating patterns of niche differentiation and interspecific interactions in species‐rich communities, as their diversity of ecological traits, behavioural plasticity and functional roles in ecosystem processes enable the integration of behavioural, spatial and community‐level perspectives on species coexistence (Chapman and Onderdonk 1998; Estrada et al. 2017; Reed and Bidner 2004).

To coexist, thrive and survive in syntopy, primates have evolved to exploit a wide spectrum of ecological niches, with community structure shaped by differences in body size, locomotion, habitat‐use, diet, ranging behaviour and social organisation (Fannin et al. 2025; Schreier et al. 2009; van Holstein et al. 2024; Youlatos and Meldrum 2011). This variation underpins niche differentiation across three primary dimensions—spatial, temporal and trophic—where overlap along one axis may be offset by segregation along another (Schoener 1974). In Neotropical primate assemblages, niche partitioning is thought to occur predominantly through differences in habitat and resource use (Schreier et al. 2009). Although previous studies have examined multiple aspects of diet and ranging (Agostini et al. 2010; Cavalcante et al. 2023; Max Snodderly et al. 2019; Stevenson et al. 2000; Veilleux et al. 2021), comparatively little attention has been directed towards sleeping sites as an important, potentially shared, resource for primates.

Sleep is a fundamental biological process that supports essential physiological and cognitive functions, but it also entails ecological costs (Reinhardt 2020). Diurnal primates spend a substantial proportion of their time sleeping (43% ± 6%; Nunn and Samson 2018), increasing their vulnerability towards predators (Barnett et al. 2012; Cheyne et al. 2013; Ellison et al. 2019). To mitigate these costs and optimise sleep quality, primates select sleeping sites based on multiple ecological factors, including predator avoidance, proximity to food resources, comfort and stability, thermoregulation and parasite avoidance (Di Bitetti et al. 2000; Dolotovskaya et al. 2026; Gazagne et al. 2025; Mekonnen et al. 2021; Savagian and Fernandez‐Duque 2017; Teichroeb et al. 2012). Recent work further suggests that lunar illumination may play an important, yet previously overlooked, role in shaping primate sleep ecology, with changes in moonlight influencing sleeping‐site selection through changes in perceived predation risk and the balance between vertical refuge and canopy cover (Zárate et al. 2026). Beyond their primary function as refuges, sleeping sites also serve as social hotspots, where settling in for the night creates opportunities for affiliative interactions between conspecifics (Anderson 1998; Brividoro et al. 2021; Loftus et al. 2024). Despite numerous studies on species‐specific sleeping site selection, their ecological role from a community perspective and the nocturnal behaviour of diurnal primates remain little understood. Understanding how sleeping sites are used within a primate community can provide insights into species' ecology, revealing patterns of interspecific competition and spatiotemporal niche differentiation.

Yet investigating sleeping‐site use at the community level has proven difficult, as simultaneously monitoring multiple arboreal species presents considerable logistical challenges (Kamilar and Beaudrot 2013). Advances in indirect and non‐invasive wildlife monitoring technologies offer new opportunities for studying tropical primates (Gazagne et al. 2025). In particular, the deployment of camera traps in sleeping site canopies offers an efficient and scalable approach to studying the behaviour of syntopic primates. While camera trapping has traditionally been applied in terrestrial contexts, its use in arboreal environments has expanded rapidly in recent years (Moore et al. 2021; Séguigne et al. 2022). Arboreal camera trapping enables continuous sampling and the detection of both diurnal and nocturnal activity (Tan et al. 2013), demonstrating strong potential for studying arboreal species, particularly primates (Bowler et al. 2017).

In the Brazilian Atlantic Forest, Carlos Botelho State Park (CBSP) hosts a unique primate community composed of southern muriquis ( *Brachyteles arachnoides* ), brown howler monkeys ( *Alouatta guariba* ), southern black‐tufted capuchins (*Sapajus nigritus cucullatus*) and black lion tamarins ( *Leontopithecus chrysopygus* ). CBSP is part of the largest remaining Brazilian Atlantic Forest remnant and is the only known location where these four species coexist (Moraes Rodrigues et al. 2016). The region has been identified as a crucial primate refuge, offering essential habitat stability in the face of ongoing environmental and climatic changes (Marques et al. 2026; Meyer et al. 2014; Pinto et al. 2023). Based on field observations and local knowledge, muriquis, howlers and tufted capuchins appeared to share and frequently use copaíba trees (*Copaifera trapezifolia* Hayne) as sleeping sites. Endemic to the Atlantic Forest, this tree species exhibits several characteristics considered important for optimal sleeping sites in these three species (Jucá et al. 2020; Mascaró Castro 2021; Zárate et al. 2026). Black lion tamarins, in contrast, typically sleep in tree hollows and occupy lower canopy strata (Albernaz 1997; Kaisin et al. 2025), and therefore do not use the canopy of copaíbas as sleeping sites. Copaíbas are emergent trees, with expansive crowns, horizontal branches and high branching points (Carvalho 2003, 2006), potentially providing stability, comfort and protection from predators (Di Bitetti et al. 2000; Mascaró Castro 2021). Furthermore, traditionally known as *pau‐óleo*, copaíbas produce an oleoresin with well‐documented anti‐inflammatory, antiparasitic, gastroprotective and antimicrobial properties (da Trindade et al. 2018; Milani et al. 2012; Teixeira et al. 2023). Given the relatively low density of old‐growth copaíbas suitable as sleeping sites at CBSP (see Methods), large individuals of this species likely represent scarce, high‐value sleeping sites subject to interspecific competition within the primate community.

This study assesses sleeping site use and nocturnal activity in a primate community using arboreal camera traps deployed in copaíbas, canopy‐dominant trees in the Brazilian Atlantic Forest. Specifically, we (1) quantify niche partitioning within the primate community and estimate spatiotemporal overlap among species; (2) assess the influence of climatic conditions and lunar illumination on sleeping site use; and (3) characterise diel activity patterns within copaíbas.

Given the similarity in sleeping‐site preferences among the primates and the relative scarcity of optimal copaíba trees, we expect copaíba trees to represent a contested resource, resulting in direct, asymmetric interspecific contest competition within the community. We further expect that, given differences in socioecology, access and priority use of copaíbas will reflect differences in species' resource holding potential (RHP; Allen and Krofel 2022)—the relative ability of a species to win direct contests over a resource. While larger groups can benefit from a numerical advantage in contests with conspecifics and heterospecifics alike (Crofoot et al. 2008; Majolo et al. 2020), larger‐bodied species tend to prevail in direct contests with smaller‐bodied ones (Ganzhorn 1999; Waser 1977). Therefore, based on the morphology and social organisation of the studied primates, as well as documented outcomes of interspecific interactions among them (Cristóbal‐Azkarate et al. 2015; Dias and Strier 2000; Falótico et al. 2021; Sobral et al. 2023), we predict the following RHP‐ranking within the community, from highest to lowest: muriquis, capuchins, howlers. Explicitly, this ranking reflects muriquis' combined size and grouping advantage, capuchins' numerical and cooperative advantage over the smaller‐grouped howlers, and howlers' comparatively limited group size despite their larger individual body mass than capuchins (see Methods).

Following the principle of complementary resource partitioning (Schoener 1974), since all three species sleep in copaíba trees, and considering sleeping sites as aggregated, non‐renewing resources, overlap along the resource axis is effectively complete. Because all three species are diurnal, complete temporal overlap in the use of copaíba trees as nocturnal sleeping sites is unavoidable, removing fine‐scale, diel temporal segregation as a viable mechanism for reducing contest competition over this resource. However, temporal segregation may still operate at coarser (e.g., weekly) temporal scales: species may reduce overlap, and thus contest competition, by differing in the periods in which they most rely on copaíba trees as sleeping sites. Given the RHP‐ranking predicted above, we expect lower‐RHP howlers to avoid copaíba use during periods of intense use by higher‐RHP syntopic primates. Muriquis and capuchins, in contrast, may be better able to reduce spatial overlap by shifting which copaíba trees they use, owing to their comparatively greater ranging patterns. Overall, we expect the observed spatio‐temporal pattern in copaíba use to be shaped by direct contest competition rather than passive, mutual niche partitioning.

Regarding abiotic factors, we predict that primates will avoid copaíbas on colder nights, favouring lower and denser canopy trees for thermoregulation (Harding et al. 2020; Qin et al. 2023). Moreover, according to the visual acuity hypothesis, we expect primates to reduce their use of copaíbas under higher lunar illumination to minimise predation risk (Zárate et al. 2026). Through arboreal camera trapping, this study advances our understanding of sleeping site selection and sleep ecology, and their role in structuring coexistence and resource partitioning within a Neotropical primate community, addressing questions that remain difficult to resolve using traditional approaches.

## 2 Methods

### 2.1 Study Area

This study was carried out in the northern part of CBSP, located along the Serra da Paranapiacaba continuum in southern São Paulo State, Brazil (24°07′53″ S, 47°56′57″ W) (Figure 1). Covering 37,644 ha of Atlantic Forest, CBSP is part of one of the most diverse biodiversity hotspots in the world (Joly et al. 2014). The Atlantic Forest is currently facing a severe biodiversity crisis resulting from large‐scale agricultural expansion, unsustainable logging and global climate change. Over the past 30 years, the Atlantic Forest has lost up to 37% of suitable habitat for species, and only about 23% of native forest cover remains (Diniz et al. 2022; Vancine et al. 2024). In this scenario, pristine protected areas such as CBSP represent important climatic refuges and havens for primates. With its unique primate community, CBSP represents an ideal setting to investigate interspecific interactions and coexistence dynamics.

**FIGURE 1:**
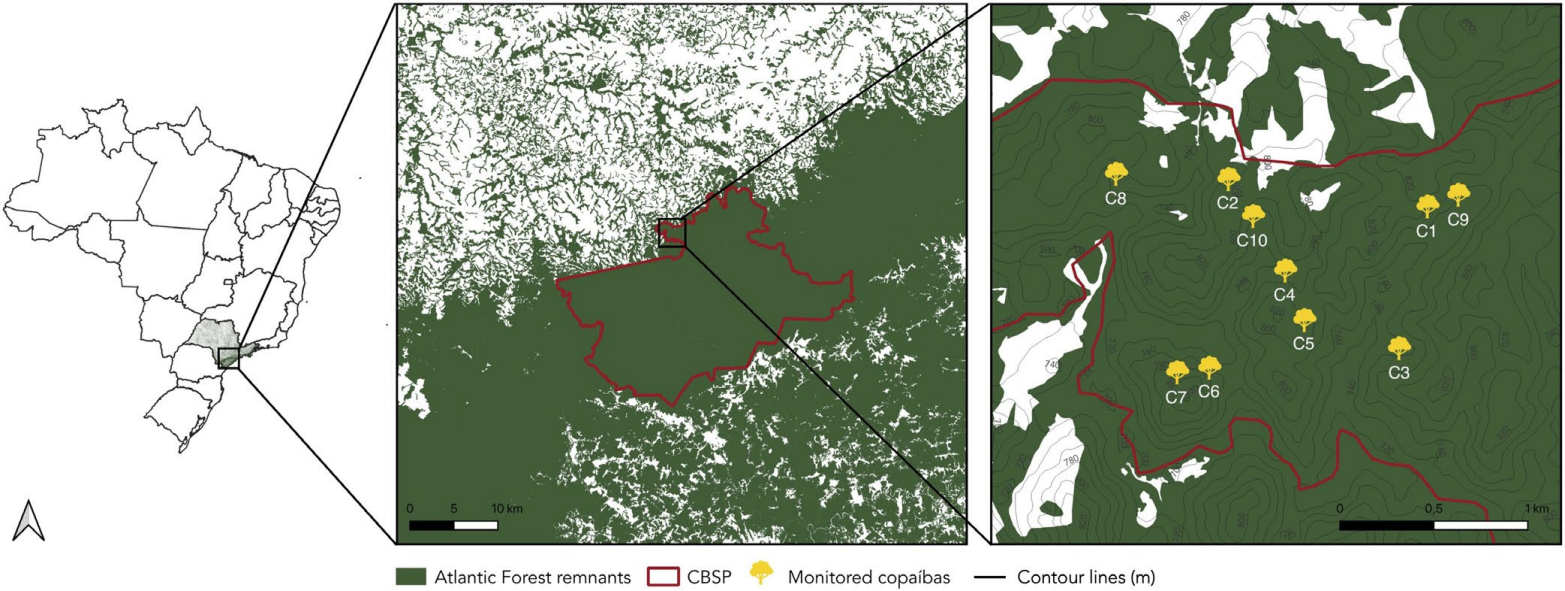
Location of the 10 monitored copaíba trees (*Copaifera trapezifolia*) situated in the northern part of Carlos Botelho State Park (CBSP; 37,644 ha), São Paulo, Brazil.

Following the Köppen climate classification, CBSP has a humid subtropical climate (Cfa), characterised by evenly distributed precipitations throughout the year and a hot summer (Kottek et al. 2006). During the study period, between December 2024 and June 2025, temperatures averaged 20.5°C, with extremes ranging from 0°C to 33.9°C (Instituto Nacional de Meteorologia, INMET). Average daily precipitation was 5 mm, ranging from 0 to 70.4 mm per day, with a cumulative total of 1057 mm during the study period (CBSP meteorological station). The study area is located at altitudes ranging from 720 to 890 m above sea level and is characterised by mountainous terrain and submontane dense ombrophilous Atlantic Forest (Florestal Fundação 2008).

### 2.2 Primate Study Species

CBSP hosts a community of four primate species: the Critically Endangered southern muriqui ( *B. arachnoides* ; Talebi et al. 2021), the Vulnerable brown howler ( *A. guariba* ; Jerusalinsky et al. 2021), the Near Threatened southern black‐tufted capuchin (*S. nigritus cucullatus*; Ludwig et al. 2022), and the Endangered black lion tamarin ( *L. chrysopygus* ; Rezende et al. 2020). Three species are well established and widely distributed throughout the protected area: *B. arachnoides* (4.11 ind. km^−2^; Bueno et al. 2013), *A. guariba* (1.86 ind. km^−2^; Sobral et al. 2023) and *S. nigritus cucullatus* (10.5 ind. km^−2^; Brocardo et al. 2010). *L. chrysopygus* was only discovered at CBSP in 2013 and has since been observed only along the park's edges and in the neighbouring Trápaga Private Natural Heritage Reserve (RPPN Trápaga), with a known population comprising a minimum of three groups of 5–7 individuals.

Regarding diet, southern muriquis and brown howlers are considered folivore‐frugivores, whereas black‐tufted capuchins have an omnivorous diet and black lion tamarins have a frugivorous‐faunivorous diet (Martins 2008; Rasec‐Silva et al. 2023; Raskin et al. 2025; Talebi et al. 2005). Body mass also differs markedly among the three species: *B. arachnoides* is the largest primate in the Americas, with adults weighing ±9 kg (Talebi and Lee 2010); *A. guariba* adults weigh approximately ±5.4 kg (Rumiz 1990); and *S. nigritus* adults weigh approximately ±2.7 kg (Fragaszy et al. 2004). *B. arachnoides* generally live in large, multi‐male, multi‐female communities of up to 54 individuals. Their fission–fusion social organisation is characterised by small mean party sizes of 3–5 individuals and larger nomadic parties comprising approximately 13–19 individuals (Coles et al. 2012). *A. guariba* typically live in groups of four to six individuals (Chaves‐Diaz et al. 2026; Jerusalinsky et al. 2021; Sobral et al. 2023). *S. nigritus* live in multi‐male, multi‐female groups, with reported group sizes ranging from 11 to 29 individuals (Izar et al. 2012; Ludwig et al. 2022). Based on our knowledge of the study area and the monitored groups, together with previous research conducted in the area (Coles et al. 2012; Sobral et al. 2023), we estimated that the area containing the monitored copaíbas was used by two capuchin groups, five howler monkey groups and one muriqui group.

### 2.3 Tree Study Species

*Copaifera trapezifolia* is a tall, slow‐growing emergent tree of the upper canopy, reaching heights of up to 30 m in mature forest (Carvalho 2003; de Souza et al. 2018). This species is an indicator of old‐growth Atlantic Forest (de Toledo et al. 2025). *C. trapezifolia* produces fruits characterised by an ovoid seed enclosed by a voluminous, bright red aril, which is known to be consumed by muriquis, howlers and capuchins (Vasquez et al. 2025; Veiga Junior and Pinto 2002). While lion tamarins are known to feed on *C. langsdorffii* (Vasquez et al. 2025), they have not been observed to feed on *C. trapezifolia*. Fruit production occurs from June to October, and copaíbas may follow a supra‐annual fruiting pattern linked to regional and climatic conditions (Carvalho 2003, 2006). This pattern has also been observed in CBSP (Coles et al. 2012), and during the study period the copaíbas did not produce fruits.

Copaíba essential oil has long been used in traditional medicine for its anti‐inflammatory, antiparasitic, gastroprotective and antimicrobial properties (da Trindade et al. 2018). The oleoresin produced by trees of the *Copaifera* genus is rich in sesquiterpene hydrocarbons (e.g., β‐caryophyllene, α‐copaene, β‐elemene, α‐humulene and germacrene D) and biologically active diterpene acids (e.g., copalic acid, kaurenoic acid, alepterolic acid and polyalthic acid), which together exhibit broad‐spectrum bioactivity against bacteria, fungi and parasites (Arruda et al. 2019; da Trindade et al. 2018; Leandro et al. 2012).

The monitored trees had previously been identified as sleeping or feeding sites for at least one of the primate species during 6 months of simultaneous behavioural data collection conducted by members of the Laboratory of Primatology at UNESP, Rio Claro, as part of parallel research projects. Between May and July and between October and December 2024, we followed groups of all four primate species, from sleeping site to sleeping site, for a total of 107 observation days, with at least 20 days per species. Based on these observations, we identified copaíbas as sleeping sites used by muriquis, howlers and capuchins and selected 10 copaíba trees for camera‐trap monitoring.

The mean nearest‐neighbour distance between monitored copaíbas was 288.7 ± 152.5 m (Figure 1). Based on our botanical survey (see Appendix S1), *C. trapezifolia* occurs at a density of 6 individuals ha^−1^, with a mean DBH of 17.7 ± 12.2 cm and a mean height of 16.5 ± 5.92 m. The monitored copaíbas were considerably larger, with a mean DBH of 67.0 ± 12.6 cm and a mean height of 25.8 ± 3.1 m, suggesting that large, old‐growth copaíbas are relatively scarce at CBSP. Notably, no *C. trapezifolia* individual comparable in size to those monitored (DBH ≥ 50 cm) was recorded in the botanical plots (maximum recorded DBH = 38.8 cm), indicating a density of large‐statured *C. trapezifolia* below 1 individual ha^−1^. Across all tree species, trees exceeding 25 m in height occurred at a density of only 6 individuals ha^−1^, while trees with a DBH greater than 65 cm occurred at a density of 20 individuals ha^−1^. Moreover, the average height of the tallest tree of any species in each plot was 18.7 ± 3.8 m, indicating that copaíbas are considerably taller and exhibit marked canopy dominance within the study area.

### 2.4 Arboreal Camera Trapping

To assess the use of *C. trapezifolia* as a sleeping site by the primate community, we installed camera traps in the canopy of 10 copaíbas within the study area (Figure 1). Data was collected from December 2024 to June 2025 over 212 days, representing a total of 4240 camera days. Due to the copaíbas' large crowns, two camera traps (Bushnell Core S 4K No Glow) were installed in each tree, each oriented towards a different zone. Camera traps were placed and oriented to monitor natural bridges between trees and/or sleeping locations, depending on the structure of each monitored tree. In each tree, at least one camera trap was oriented towards the principal observed access route to record primates entering and leaving the tree. Camera placement and orientation considered sun exposure, wind and potential vegetation disturbance. Before the main monitoring period, we conducted a pilot study between October and December 2024 to refine camera‐trap installation, improve detectability and reduce false triggers. During this period, we tested different camera positions and angles. Because the monitored copaíbas were emergent trees with relatively few potential access points, cameras could be positioned strategically to maximise the detection of primate movements through these routes.

Installed at an average height of 24.2 ± 3.7 m, camera traps were attached to large branches using buckle straps to withstand weather conditions. Camera traps operated continuously and were set up to record 30‐s videos upon each trigger, with a 10‐s delay between consecutive recordings. Sensor sensitivity was set to low or medium depending on canopy configuration to ensure detection of primates within the camera's range while reducing false triggers. Once a month, we climbed the trees using a single‐rope technique to exchange SD cards (256 GB; a large capacity preferred in canopy settings given the high number of false triggers) and replace camera‐trap lithium batteries if necessary. These maintenance visits involved approximately 80 tree climbs over the study period.

### 2.5 Data Analysis

All videos were processed using *Timelapse* software (Greenberg et al. 2019). For each video, we recorded the date and time, primate species, number of individuals, sex–age categories and main behaviour (see Section 2.5.3). We estimated the minimum number of individuals of each species recorded at each monitored copaíba by calculating the maximum number of individuals observed in a single detection, both overall and independently for each sex–age category.

Based on the camera‐trap footage, four types of data were used in the statistical analyses: detections, occurrences, visits and nights. A *detection* was defined as each camera‐trap trigger resulting in a 30‐s video showing an animal. An *occurrence* was defined as all detections of a given species pooled from across the two camera traps at a given tree within the same 15‐min time bin. This approach prevented overestimation of primate presence resulting from the dual camera setup installed in each tree and reduced bias associated with behavioural detectability. These occurrences allowed us to assess the temporal use of *C. trapezifolia* by each species. Detections and occurrences were classified as diurnal or nocturnal based on sunset and sunrise times calculated for the study area using the *suncalc* package (version 0.5.1; Thieurmel and Elmarhraoui 2017), based on the geographic coordinates of the study site.

A *visit* was defined as a sequence of consecutive detections of a given species at a given copaíba, representing a continuous period of tree use. Visit duration was calculated as the time interval between the first and last detection, with an additional 30 s added to account for the last detection. When the time lapse between two detections exceeded 15 min, they were considered two distinct visits. This conservative threshold assumed that if no detections were recorded for more than 15 min, the species may have left and subsequently returned to the tree, therefore accounting for two distinct visits. Visits were used to calculate behavioural activity budgets for each primate species while in the copaíbas (see Section 2.5.3).

A *night* was defined as a night spent by a primate species in a copaíba. To determine whether a species had slept in one of the trees, we considered two conditions: either (1) a detection occurred between sunset and sunrise or (2) detections occurred within both species‐specific sleeping site entry (evening) and exit (morning) time windows. We defined these time‐windows based on the direct observations of the four primate species during the simultaneous daily follows.

#### 2.5.1 Temporal Overlap

To assess how primates temporally segregated their use of copaíbas, we quantified overlap at two temporal scales: fine‐scale (diel) overlap and coarse‐scale (weekly) overlap based on occurrence data—detections aggregated in 15‐min time bins. For diel activity patterns, we converted recorded time into solar time to eliminate the influence of seasonal variations in day length and subsequently transformed solar time into radians (0–2π) to account for the circular nature of the 24‐h cycle (Nouvellet et al. 2012). Diel temporal overlap between species pairs was then estimated using the *overlap* package in R (version 0.3.9; Meredith and Ridout 2014), which applies kernel density estimates (KDE) to calculate the Δ_4_ overlap index. This index ranges from 0 (no overlap) to 1 (complete overlap), with Δ_4_ > 0.75 often indicating high overlap and Δ_4_ < 0.5 indicating low overlap (Wu et al. 2025).

To assess weekly temporal overlap among the primate species, we calculated a weekly relative abundance index (RAI) using occurrence counts (Liu et al. 2013). For each species and week, defined as consecutive 7‐day sampling periods, the number of independent occurrences was summed and standardised by the number of active camera‐trap days per week. Weekly RAI values were then used to calculate pairwise temporal overlap using Schoener's index (Schoener's *D*; Schoener 1968). This index ranges from 0 (no overlap) to 1 (complete overlap) and estimates similarity in weekly occurrence patterns (Santos et al. 2013; Schoener 1974). Finally, to assess weekly overlap specifically in the use of copaíbas as sleeping sites, we calculated weekly RAIs from nights when copaíbas were used as sleeping sites (i.e., a single record per species per night) and calculated pairwise overlap using Schoener's *D* (Schoener 1968).

#### 2.5.2 Spatial Overlap

Pairwise spatial overlap among species was calculated using Pianka's overlap index (Pianka's *O*), which ranges from 0 (no overlap) to 1 (complete overlap) and quantifies the degree of overlap in resource use between each pair of species (Pianka 1973). Spatial overlap was calculated based on the proportional use of individual copaíba trees, with each tree treated as a discrete spatial resource unit, and species occurrence data used as the measure of use intensity. To illustrate the spatial activity patterns of each primate species across the 10 monitored copaíbas, we generated kernel density estimates (KDE) using the kernelUD function from the adehabitatHR package in R (Calenge 2011), based on georeferenced copaíba locations weighted by species occurrence data. Spatial distributions were visualised using 95% and 50% isopleths, representing the broad use area and core use area, respectively. To illustrate the spatial occurrence patterns of each primate species in the 10 copaíbas, we generated 95% and 50% KDEs using the *KernelUD* function (*adehabitatHR* R package version; Calenge 2011), based on all occurrences and the geographic coordinates of each copaíba.

#### 2.5.3 Impact of Abiotic Variables on Sleeping‐Site Selection

To assess the influence of abiotic environmental factors on the use of copaíbas as sleeping sites by the primate community, we considered minimum temperature, mean wind speed, total precipitation and moonlight on the day of sleeping‐site entry. Climatic variables were obtained from the meteorological station at CBSP. Moonlight was quantified using an effective moonlight index combining lunar illumination fraction and angular elevation above the horizon, calculated with the *suncalc* package (version 0.5.1; Thieurmel and Elmarhraoui 2017). For each species, we fitted a binomial generalised linear model (GLM) in which each day was classified based on the presence (=1) or absence (=0) of primates during the night. To test for multicollinearity among predictors, we calculated the variance inflation factor (VIF) and all variables retained in the model had a VIF ≤ 2.5 (Johnston et al. 2018). Model selection was performed using a bidirectional stepwise procedure based on Akaike's information criterion (AIC), and the model with the lowest AIC was retained as the best‐supported model (Burnham and Anderson 2004). The significance of the selected models was evaluated by comparing them to null models using likelihood ratio tests. For predictors retained in the selected models, parameter estimates and their 95% confidence intervals were reported to describe the direction and uncertainty of their effects. Model assumptions and goodness‐of‐fit were assessed using simulated residuals implemented in the *DHARMa* package (version 0.4.7; Hartig 2016).

#### 2.5.4 Behavioural Analysis

To assess the diurnal behavioural activity of primates in the copaíbas, each diurnal 30‐s video was treated as a scan sample, and we recorded the predominant group behaviour when more than one individual appeared in the video (Altmann 1974). Behavioural categories included foraging, moving, travelling, resting, playing and inactive (see Table S1). These data were used to explore diurnal activity during visits. For each visit, the proportion of scans assigned to each behavioural category was calculated by dividing the number of scans of a given behaviour by the total number of scans recorded during that visit. Time spent in each behaviour was then estimated by multiplying this proportion by the total visit duration. Activity budgets were then calculated for each species by summing the time allocated to each behavioural category across all visits and copaíbas. This approach, based on scaling behavioural proportions by visit duration rather than raw detections, reduced bias associated with unequal detectability among behaviours by preventing sequences of repeated detections from disproportionately inflating their contribution to diurnal activity budgets.

To assess nocturnal activity, behaviour was analysed at the night level rather than at the visit level, because even without being detected—due to a lack of movement sufficient to trigger camera traps—primates remained in the tree. Night duration was determined based on sunset and sunrise times. To estimate the proportion of time primates were awake during the night, we classified behaviours recorded in detections into two categories: a sleeping state and an awake state (all other behaviours, including resting with eyes open). Individuals were considered asleep if, throughout the detection (30 s), they exhibited relaxed musculature and, when visible, closed eyes (Roach et al. 2025). For each visit, a behaviour‐specific buffer was added to the time of the last detection to account for the transition to sleep: sleeping had a 0‐min buffer, resting had a 5‐min buffer and all other active behaviours had a 15‐min buffer. Given the scarcity of available data on sleep onset latencies in wild primates, these buffers were estimated based on research on sleep latency in macaques (*Macaca* spp.), the most studied primate genus in this regard (Davin et al. 2022; Ishikawa et al. 2017; Roach et al. 2025). Time between detections, except during defined buffer periods, was considered as sleeping. We also compared activity patterns within twilight periods, defined as the periods after sunset and before sunrise when the sun is 0°–6° below the horizon and some solar illumination remains (Tan et al. 2013), and within core nighttime, defined as the portion of the night excluding twilight periods.

Although no copaíba exudate was observed on the branches, primates were occasionally observed rubbing their fur against them. We therefore recorded all occurrences of fur‐rubbing that differed from anogenital scent‐marking behaviours, including the behavioural context, body region involved and time of day. These observations were used to characterise the contexts and body regions associated with fur‐rubbing across species.

## 3 Results

In total, 29,259 camera triggers were recorded, of which 4289 (15%) contained animal detections, with the remaining 85% representing false triggers. Of the detection‐positive videos, 4195 included primates and 94 featured other species—seven mammal species and at least nine bird species (Table S2). Detections of other mammal species were sporadic and did not involve interactions with primates. Over the 7‐month data collection period, we recorded 1253 primate visits to copaíbas (Videos S1–S4), including 836 by muriquis, 252 by howlers and 165 by capuchins; black lion tamarins were not observed in the copaíbas. Muriquis visited most regularly (every 1.12 ± 0.49 days), followed by howlers (every 1.67 ± 1.08 days) and capuchins (every 2.28 ± 1.93 days). Primates slept in the monitored copaíbas on 158 occasions (tree‐night records), across 115 distinct nights: muriquis on 94 tree‐nights across 9 trees, howlers on 22 across 9 trees and capuchins on 42 across 7 trees (Figure 2; Table S3).

**FIGURE 2:**
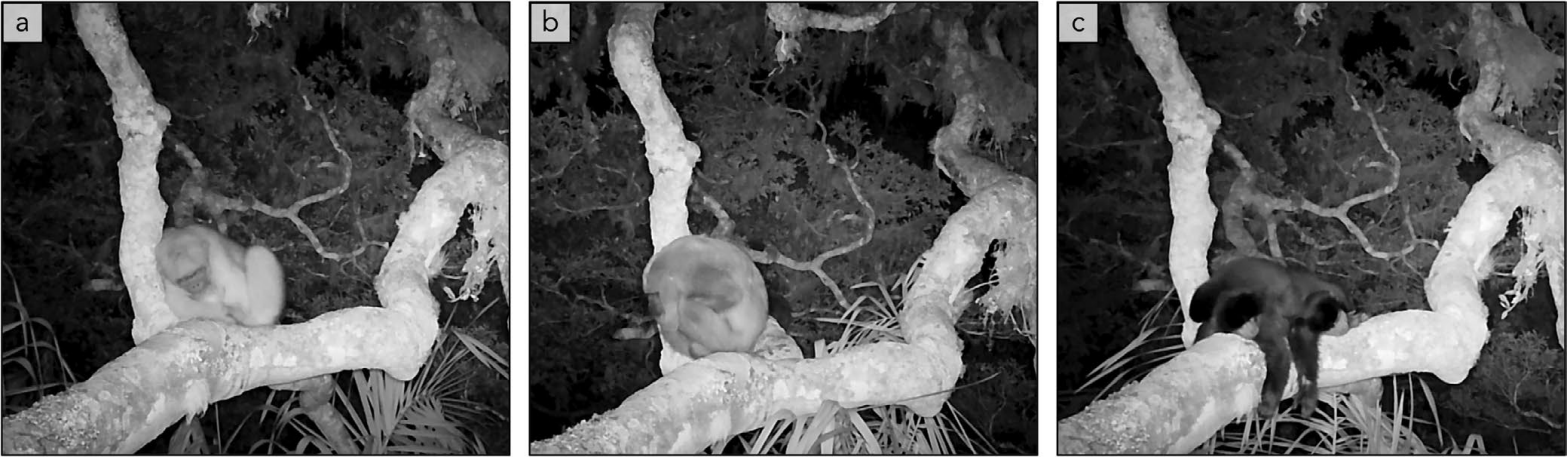
Camera‐trap images showing primates sleeping on the same branch of the same copaíba (*Copaifera trapezifolia*) tree (C2) on different nights: (a) *Brachyteles arachnoides* , (b) *Alouatta guariba* and (c) *Sapajus nigritus cucullatus*.

The mean number of individuals per video was 1.92 ± 1.19 for muriquis, 2.41 ± 1.46 for howlers and 2.37 ± 1.65 for capuchins (mean ± SD). The maximum number of individuals recorded in a single video was 9 for muriquis, 6 for howlers and 9 for capuchins (Table S4). Across monitored trees, the mean minimum number of individuals recorded was 10.60 ± 3.17 for muriquis, 4.70 ± 1.34 for howlers and 6.78 ± 4.29 for capuchins (mean ± SD; Table S4). These estimates were based on the observed sex–age categories and the maximum number of individuals recorded in a single detection. Because individuals could not be reliably identified across videos, these values represent conservative minimum estimates of the number of individuals recorded at each tree, rather than confirmed group sizes or group compositions.

### 3.1 Temporal Overlap

Diel activity patterns showed morning and evening peaks for all three species, corresponding to emergence from and entry into sleeping sites at dawn and dusk, consistent with direct behavioural observations (Figure 3). Capuchins showed low daytime activity and sharp peaks at sunrise and sunset, suggesting copaíbas are used primarily as sleeping sites. Howlers showed higher daytime activity, using copaíbas frequently in the morning after waking and as resting sites throughout the day. Muriquis' activity remained relatively stable throughout the day, suggesting that these trees are potentially incorporated into their daily routes and used for purposes beyond sleeping, including daytime resting. Pairwise Δ_4_ coefficients indicated stronger diel overlap between muriquis and howlers (Δ_4_ = 0.76) than between either species and capuchins (muriquis–capuchins: Δ_4_ = 0.69; howlers–capuchins: Δ_4_ = 0.52).

**FIGURE 3:**
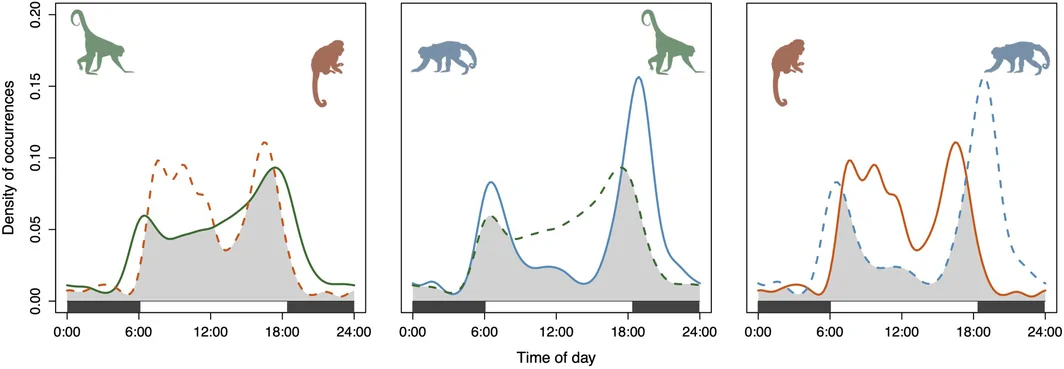
Comparison of diel activity patterns among primate species based on camera trap occurrences in copaíba trees (*Copaifera trapezifolia*). Kernel density estimates of activity are shown for each species pairwise comparison: Muriquis ( *Brachyteles arachnoides* ; green), howlers ( *Alouatta guariba* ; red) and capuchins (*Sapajus nigritus cucullatus*; blue). Solid and dashed lines represent the activity distributions of each species.

Weekly temporal overlap among species, quantified using Schoener's *D* based on weekly RAI, was generally high but varied among pairs. Overlap was greatest between muriquis and capuchins (*D* = 0.75) and between muriquis and howlers (*D* = 0.74), whereas howlers–capuchins showed lower, although still substantial, overlap (*D* = 0.62).

At the species level, muriquis most frequently slept in monitored copaíbas (every 2.61 ± 3.27 nights; *n* = 78), followed by capuchins (every 4.89 ± 4.74 nights; *n* = 37), whereas howlers used them less frequently (every 8.9 ± 11.2 nights; *n* = 21). Although group identity could not be established from the camera‐trap footage, primate species were observed sleeping in the same copaíba trees on consecutive nights. Muriquis most frequently showed this pattern (*n* = 26), with sequences lasting up to four nights. In contrast, this was observed only once in howlers and six times in capuchins, with sequences limited to two consecutive nights. Over 36 nights, different primate species, groups and/or subgroups used multiple monitored trees on the same night (Table S5). These observations involved two different species on 14 nights, distinct groups or subgroups of the same species on 22 nights, and both patterns on seven nights. Although no video recorded two species sleeping simultaneously in the same copaíba, the timing of the detections suggested that two species may have used the same copaíba as a sleeping site on three nights, with one event recorded for each species pair. Considering independent sleep events, howlers appeared to reduce their use of copaíbas when muriquis and capuchins showed higher levels of use, and vice versa (Figure 4). This pattern was consistent with lower weekly night‐use overlap between muriquis and howlers (*D* = 0.42) and between howlers and capuchins (*D* = 0.43), whereas weekly night‐use overlap was higher between muriquis and capuchins (*D* = 0.79).

**FIGURE 4:**
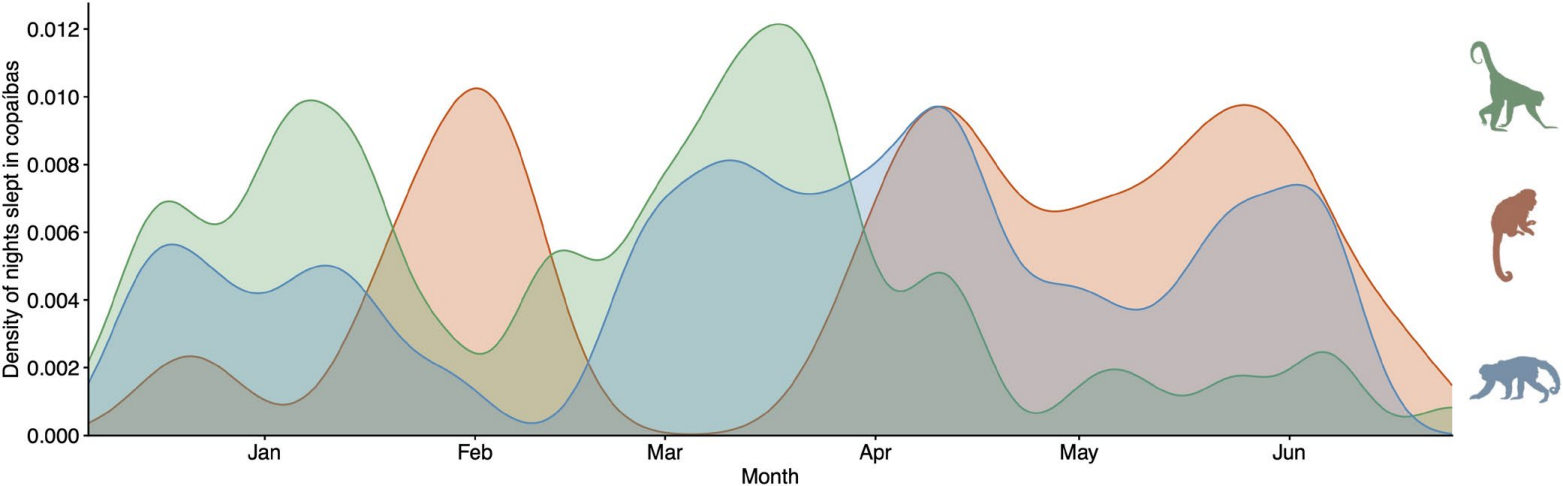
Use of copaíba trees (*Copaifera trapezifolia*) as sleeping sites during the 7‐month study period by the primate species: Muriquis ( *Brachyteles arachnoides* ; green), howlers ( *Alouatta guariba* ; red) and capuchins (*Sapajus nigritus cucullatus*; blue). Density curves represent weekly Relative Abundance Index (RAI) of nights slept in copaíbas by each species (one record per species per night), smoothed using kernel density estimation.

### 3.2 Spatial Overlap

Spatial use differed among species, with each species using different copaíbas most intensively (Figure 5). Pianka's *O* indicated low overlap between muriquis and capuchins (*O* = 0.17), higher overlap between howlers and muriquis (*O* = 0.73) and moderate overlap between howlers and capuchins (*O* = 0.57).

**FIGURE 5:**
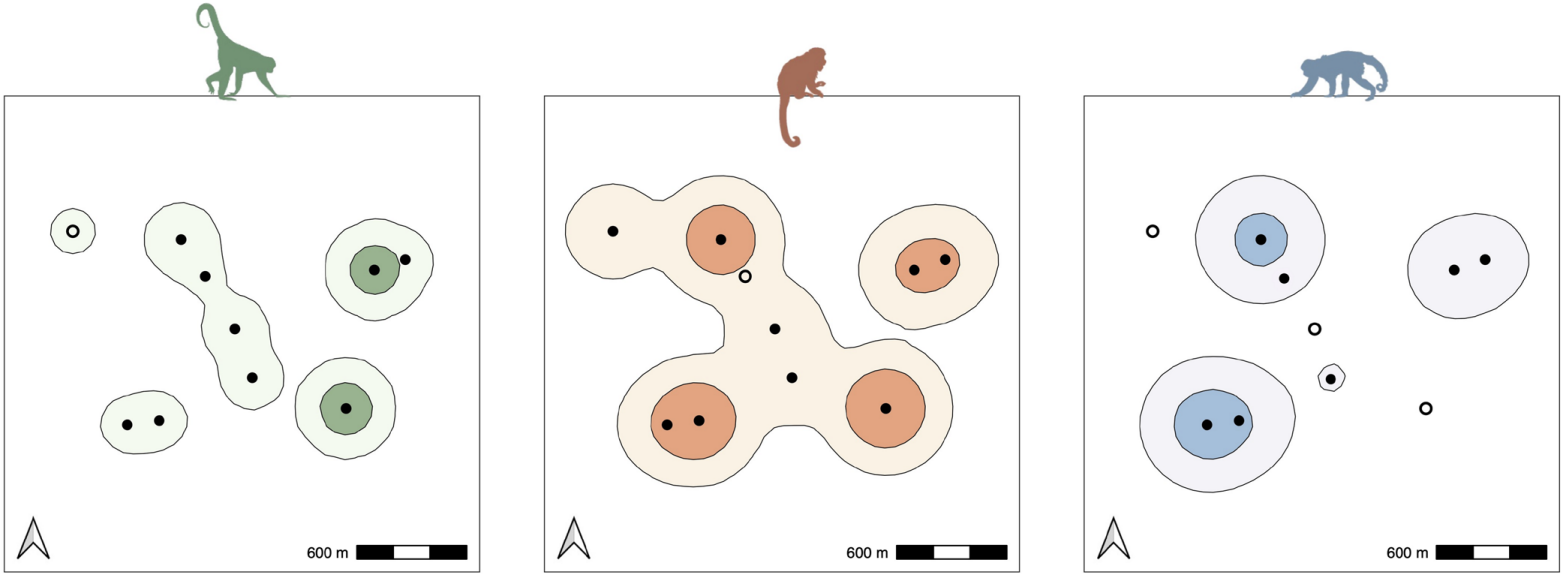
Kernel density estimates illustrating the 50% (dark) and 95% (light) isopleths of spatial use for each primate species: Muriquis ( *Brachyteles arachnoides* ; green), howlers ( *Alouatta guariba* ; red) and capuchins (*Sapajus nigritus cucullatus*; blue). Copaíba trees (*Copaifera trapezifolia*) used as sleeping sites are indicated by filled black circles, whereas non‐sleeping site trees are shown as open circles.

### 3.3 Impact of Abiotic Variables on Sleeping‐Site Selection

For muriquis, the best‐supported model included minimum temperature, average wind speed and moon phase (Table S6). Minimum temperature was positively associated with sleeping‐site use (β = 0.242, 95% CI [0.113, 0.372]; Figure 6), while average wind speed showed a positive but uncertain association, with the 95% confidence interval including zero (β = 0.373, 95% CI [−0.010, 0.755]; Figure 5). Moon phase was negatively associated with sleeping‐site use (β = −1.189, 95% CI [−2.370, −0.008]; Figure 6). For capuchins, the best‐supported model included minimum temperature and moon phase (Table S6). Minimum temperature was positively associated with sleeping‐site use (β = 0.176, 95% CI [0.026, 0.326]; Figure 5), whereas the negative association with moon phase was uncertain, as its 95% confidence interval included zero (β = −1.410, 95% CI [−2.834, 0.015]; Figure 6). For howlers, the null model was the best‐supported model (Table S6), indicating no support for an effect of the abiotic variables considered. Precipitation was not retained in the best‐supported model for any species.

**FIGURE 6:**
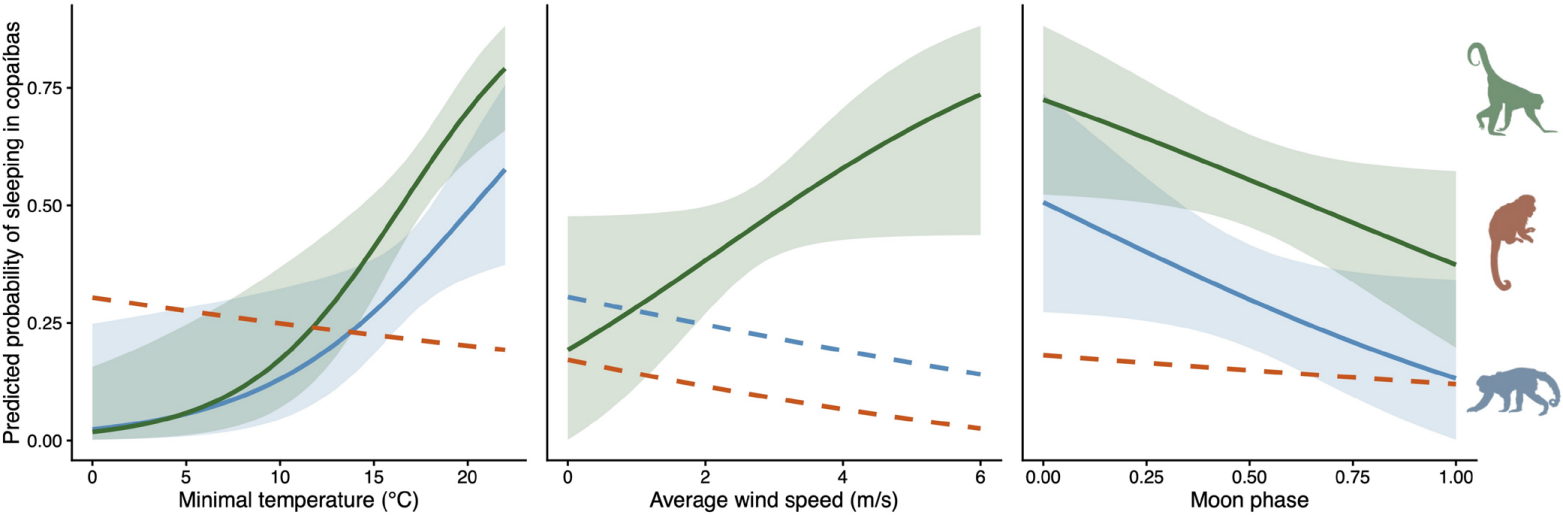
Predicted probabilities (±95% confidence intervals) of nocturnal presence in copaíba trees (*Copaifera trapezifolia*) for each primate species—muriquis ( *Brachyteles arachnoides* ; green), howlers ( *Alouatta guariba* ; red) and capuchins (*Sapajus nigritus cucullatus*; blue)—derived from binomial generalised linear models. Dotted lines represent predicted probabilities for variables that were not retained in the species‐specific best models.

### 3.4 Behavioural Analysis

Diurnal activity budgets within copaíbas were dominated by resting, travelling and feeding across all three species (Figure S2; Video S1). Moving and travelling dominated daytime activity in capuchins (67.3%; n_daytime_ = 371 detections) and muriquis (51.4%), with muriquis also spending substantial time resting (38.0%; n_daytime_ = 2358 detections). Howlers, in contrast, rested more (62.8%) than they travelled (30.4%; n_daytime_ = 680 detections). Camera‐trap videos detected nocturnal activity by all three species (Video S2). Behavioural activity recorded in the videos differed between the twilight buffer and the core nighttime period (Figures S3 and S4). During twilight, howlers were predominantly moving (68.8%; n_twilight_ = 7 detections), whereas muriquis spent most of the recorded detections resting (45.4%; n_twilight_ = 179 detections). Capuchins were mainly in movement (34.5%) and travelling (30.8%; n_twilight_ = 123 detections). During core nighttime, howlers and muriquis were predominantly resting (70.2% and 63.7%, respectively; n_core_ = 57 and 492 detections, respectively), whereas capuchins were primarily travelling (48.2%) and moving (35.6%; n_core_ = 238 detections).

On nights spent in copaíbas, activity peaks were strictly concentrated around sunrise and sunset (Figure 7). Howlers left after sunrise and entered before sunset. Muriquis and capuchins began to wake up slightly before sunrise, with peak activity coinciding with sunrise, capturing them leaving the sleeping sites. While muriquis started entering the sleeping sites around an hour before sunset, capuchins showed peak activity after sunset, around the end of dusk. Capuchins were the most active during twilight (51.9%; 24.5 ± 11.5 min), followed by muriquis (41.0%; 19.4 ± 8.9 min) and howlers (26.7%; 13 ± 6.9 min). The same pattern was observed during core night‐time: capuchins (6.6%; 44.6 ± 42.6 min), muriquis (6.6%; 44.4 ± 55.4 min) and howlers (3.88%; 28.4 ± 23.4 min). Overall, muriquis and capuchins remained more active at night, partially linked to their later sleeping times (Figure 7).

**FIGURE 7:**
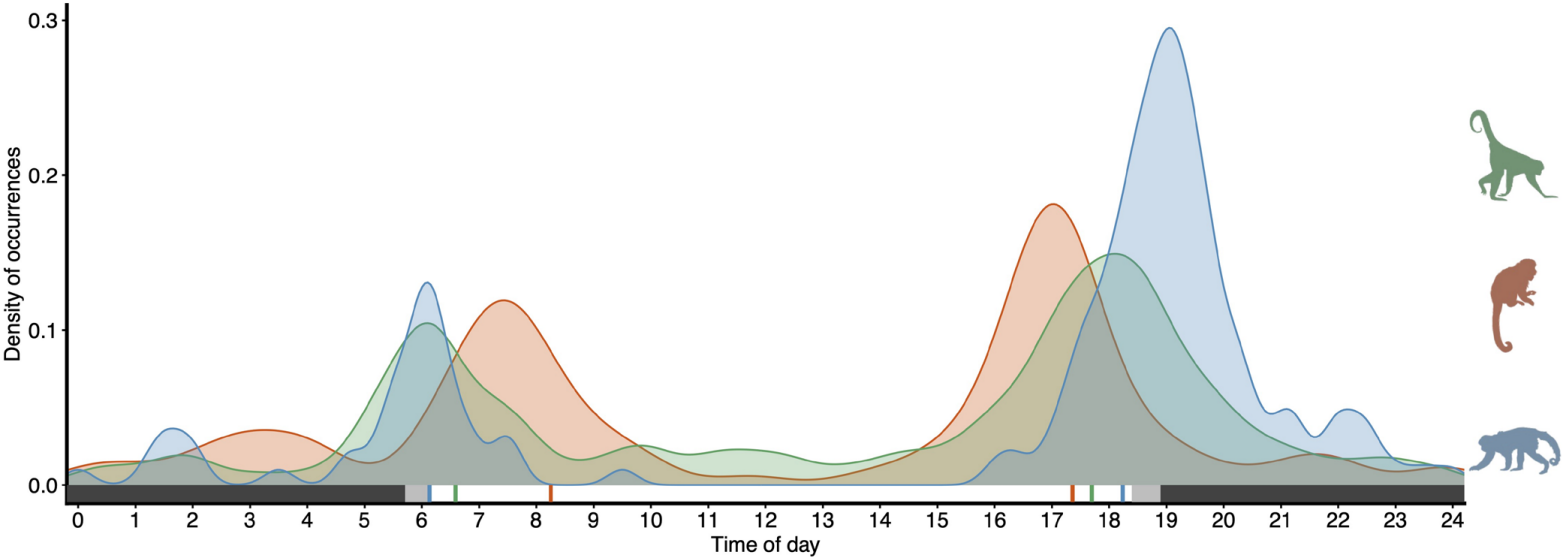
Diel distribution of occurrences in copaíba trees (*Copaifera trapezifolia*) used as sleeping sites by primates: Muriquis ( *Brachyteles arachnoides* ; green), howlers ( *Alouatta guariba* ; red) and capuchins (*Sapajus nigritus cucullatus*; blue). Observations when groups were asleep were excluded. For comparison, solid vertical lines represent species‐specific mean entry and exit times obtained from direct observations. Twilight and core nighttime periods are shown in grey and black, respectively.

We recorded 13 interspecific encounters within the monitored copaíbas: 10 muriqui‐howler, 2 muriqui‐capuchin and 1 howler‐capuchin (Figure S1; Table S7). Based on the time of day, five of these encounters appeared to occur when the primates may have been settling in for the night. In nine encounters, howlers were already present in a monitored copaíba when muriquis subsequently arrived, after which the muriquis remained in the tree. Five encounters were also directly captured on video; four were agonistic muriqui‐howler interactions, with muriquis chasing howlers out of the trees and, on one occasion, grabbing a juvenile howler (Video S3). The single howler–capuchin encounter was non‐agonistic, with neither species demonstrating a response to the other's presence (Video S3).

Fur‐rubbing was observed in all three primate species across 76 videos: muriquis (*n* = 23), howlers (*n* = 47) and capuchins (*n* = 6). Across species, individuals predominantly rubbed the dorsal body region against copaíba branches in repeated side‐to‐side movements (Video S4). Howlers and muriquis exclusively rubbed their dorsal region, whereas capuchins also rubbed their chest and neck. Based on species‐specific waking and sleeping times, 52.6% of fur‐rubbing videos were recorded during the species‐specific sleeping period, whereas 47.4% occurred during the active period. The proportion of videos recorded during the sleeping period was 55.3% for howlers (*n* = 26), 39.1% for muriquis (*n* = 9) and 83.3% for capuchins (*n* = 5). Fur‐rubbing was recorded while primates were moving in 42 videos (55.3%), resting in 19 videos (25.0%) and travelling in 15 videos (19.7%).

## 4 Discussion

Sleeping sites are often overlooked in studies of primate communities, yet our results suggest that they may represent important, limited resources shared among syntopic primates, revealing patterns of temporal and spatial partitioning. Using arboreal camera trapping, we show that three primate species frequently sleep in *C. trapezifolia* trees, highlighting their importance as key sleeping sites in an Atlantic Forest primate community and providing new insights into patterns of spatiotemporal partitioning. Despite substantial overlap in use, species differed in when and which trees they visited, reflecting species‐specific strategies of sleeping‐site selection and responses to abiotic factors such as moonlight and temperature. Continuous canopy monitoring captured behaviours that are rarely observed using traditional methods, including previously undocumented nocturnal activity, thereby providing new insights into sleeping‐site use and sleep ecology in arboreal primates.

Sleeping‐site selection is broadly similar across muriquis, howlers and capuchins. These species prefer tall, emergent trees with large crowns and specific structural traits, consistent with predator avoidance, as well as other functions such as thermoregulation, parasite deterrence, social contact and proximity to feeding sites (Brividoro et al. 2019; Di Bitetti et al. 2000; Jucá et al. 2020; Mascaró Castro 2021). *Copaifera trapezifolia* exhibits key structural and ecological characteristics that make it a highly suitable and contested sleeping‐site. These trees were intensively used by the primate community throughout the study period, with recorded primate presence on more than 50% of the monitored nights.

By considering copaíbas as a shared resource and applying concepts of niche overlap, we observed spatiotemporal partitioning among species. Muriquis and capuchins showed high temporal overlap in nocturnal copaíba use but predominantly selected different trees as sleeping sites, resulting in very low spatial overlap. In contrast, howlers showed greater spatial overlap with both muriquis and capuchins, but lower weekly temporal overlap in night‐use. Together, these results indicate that species reduce direct competition by partitioning sleeping‐site use across dimensions, with temporal overlap offset by spatial segregation, and vice versa.

These patterns are likely driven by interspecific differences in ranging behaviour and competitive ability (i.e., RHP). Based on the observed sex–age categories and ranging patterns of howler monkey groups in the area (Sobral et al. 2023), we estimated that the monitored copaíba trees were used by at least five distinct howler groups. Their restricted ranging patterns may limit their access to alternative copaíbas when other species occupy preferred sites (Chaves‐Diaz et al. 2026; Sobral et al. 2023). Moreover, Moreover, howlers are often harassed or dominated by syntopic primates, being chased and forced to modify their route, as observed here (Cristóbal‐Azkarate et al. 2015; Falótico et al. 2021; Sobral et al. 2023). Therefore, despite entering sleeping sites earlier than the other species, early arrival may not guarantee continued access to these sites. Indeed, interspecific encounters recorded in monitored copaíbas indicate that muriquis can subsequently occupy and remain in a tree initially occupied by howlers, corroborating our predicted RHP ranking: the higher‐RHP muriquis displaced the lower‐RHP howlers. In our study, aside from a single neutral encounter with capuchins, all heterospecific encounters between howlers and muriquis resulted in howlers leaving the copaíba trees. Under these conditions, the lower nightly‐use overlap between howlers and the other species may reflect temporal differences in copaíba use that reduce the likelihood of potentially costly encounters. In contrast, muriquis and capuchins—the two higher‐RHP species—have larger home ranges (Coles et al. 2012; Dias and Strier 2003; Presotto and Izar 2010), potentially providing greater flexibility to spatially segregate their use of copaíba trees and select these trees as sleeping sites under preferred conditions.

High temporal overlap in nightly‐use between muriquis and capuchins likely reflects convergence in sleeping‐site selection, with both species preferring copaíba trees under similar abiotic conditions—warmer nights and lower moonlight. Thermoregulation is a key factor influencing sleep ecology in primates and other homeothermic animals (Downs et al. 2015; Harding et al. 2019, 2020; Pozzi et al. 2022). Consistent with this, muriquis and capuchins seem to preferentially use higher, more open canopy trees such as copaíbas on warmer nights, while favouring lower, denser canopy structures under colder conditions. Moreover, capuchins at CBSP often use the palm leaves of *Attalea dubia* as sleeping sites when night temperatures drop (Fogaça 2014). According to the social thermoregulation hypothesis, primates may select sleeping sites with more branches and denser canopies to increase sleeping cluster size on cold nights (Brividoro et al. 2023; Mekonnen et al. 2021; Qin et al. 2023; Smith et al. 2018). Moonlight has rarely been explored as a potential factor influencing sleep quality and sleeping‐site selection in primates (Zárate et al. 2026). Following the visual acuity hypothesis, muriquis and capuchins may favour copaíbas on darker nights to seek vertical refuge under higher perceived predation risk (Bischof et al. 2024; Prugh and Golden 2014). Both species may also avoid emergent, open‐canopy trees on nights with high lunar illumination, as this may increase nocturnal activity and impair sleep quality (Ayers et al. 2020; Reyes et al. 2021; Spaan et al. 2026). While rainfall did not appear to affect sleeping‐site selection, muriquis used copaíba trees more frequently on windy nights, potentially due to the structural stability of these towering trees. The absence of detectable abiotic effects on copaíba use by howlers—despite documented evidence to the contrary (Brividoro et al. 2023; Contreras et al. 2025)—suggests that competition with syntopic primates may limit their ability to use these trees under preferred conditions.

While the intensive reuse of sleeping sites across multiple nights is not fully consistent with the parasite avoidance hypothesis (Di Bitetti et al. 2000; Hausfater and Meade 1982), the well‐known pharmaceutical properties of copaíbas may offer an additional and complementary explanation for their strong preference by all three species. The oleoresin produced by *Copaifera* species is well documented for its antimicrobial, antiparasitic and insecticidal properties (da Trindade et al. 2018; Leandro et al. 2012). Sleeping in close contact with copaíba bark—and occasionally rubbing against it, as observed on camera‐trap footage—may therefore offer passive chemical protection against ectoparasites and pathogens. Rather than spatial parasite avoidance, this pattern may represent a form of prophylactic zoopharmacognosy, a behaviour documented in other Atlantic Forest mammal communities (Kaisin et al. 2022). While this hypothesis remains to be tested directly—for example, by analysing the chemical composition of the bark, assessing parasite abundance, and comparing fur‐rubbing rates and body regions rubbed on copaíba versus other tree species—it raises the intriguing possibility that the potential pharmacological properties of copaíbas may also influence sleeping‐site selection. Importantly, however, we expect primates to primarily select copaíbas for their structural properties, including their height, canopy architecture and stability, while potential pharmacological properties may represent an additional benefit.

By capturing primates entering and leaving their sleeping sites, arboreal camera trapping also revealed diel activity patterns—consistent with those obtained through traditional behavioural methods (Chaves‐Diaz et al. 2026; Izar et al. 2012; Talebi and Lee 2010)—highlighting interspecific differences in behaviour and ecology. While capuchins used copaíbas almost exclusively as sleeping sites, muriquis and howler monkeys also visited these trees during the day, often as resting sites, resulting in greater diel overlap between the latter species. These patterns may reflect interspecific differences in both time allocation linked to diet and vertical space use linked to caudal morphology. Given their partially folivorous diets, both muriquis and howlers are expected to favour reduced energetic expenditure and extended periods of resting, consistent with an energy‐saving strategy (Chaves‐Diaz et al. 2026; Dias and Rangel Negrín 2026; Milton 1981; Talebi and Lee 2010). As atelids possess fully prehensile tails that facilitate movement in the upper canopy, muriquis and howlers exploit higher forest strata and thus more frequently incorporate emergent trees, such as copaíbas, into their daily routes (Iurck et al. 2013; Johnson et al. 2023; Naas et al. 2025). On the other hand, capuchins' semi‐prehensile tails may limit their use of the higher canopy strata, favouring lower‐ and mid‐canopy strata (Mangalam et al. 2022; Mendoza‐Soto et al. 2024).

Our camera trap data revealed that all three syntopic primates aligned their sleeping times with sun cycles, adopting varying strategies and utilising daytime as an ecological resource (Sanchez et al. 2022; Santini et al. 2015). Muriquis and capuchins timed sleeping‐site entry and exit closely with sunset and sunrise, respectively, likely balancing foraging opportunities and safety, as observed in other primate species (José‐Domínguez et al. 2015; Mekonnen et al. 2021; Pozzi et al. 2022). Capuchins appeared to enter their sleeping sites after dusk, suggesting a tendency to remain active under low light conditions and thereby maximise late‐day foraging opportunities—a pattern consistent with their energy‐maximising strategy (Gómez‐Posada et al. 2019) and also reported in snub‐nosed monkeys ( *Rhinopithecus brelichi* ; Tan et al. 2013). In contrast, howlers typically entered and exited sleeping sites about 1 h before sunset and emerged over 1 h after sunrise, spending longer periods in these trees, consistent with an energy‐saving strategy (Jucá et al. 2020; Rangel‐Negrín et al. 2018). Since primates living in smaller groups may be more vulnerable to predation (Hill and Lee 1998; van Schaik 1983; van Schaik et al. 1983), howlers may also enter sleeping sites earlier to reduce their detectability by crepuscular predators (Dolotovskaya et al. 2026; Hasan et al. 2025; José‐Domínguez et al. 2015).

At night, camera traps revealed unprecedented nocturnal and sleep‐related behaviours, highlighting the strong potential of this method for studying sleep ecology in wild primates. Twilight emerged as the most behaviourally active period across all three species—capturing the primates positioning themselves in the trees and settling into sleep—with capuchins showing the highest activity levels, followed by muriquis and howlers. This species‐level ranking was maintained during the core nighttime period, though overall activity dropped markedly. Critically, nocturnal videos revealed that activity was not simply residual wakefulness at the limits of the night: individuals of all three species were observed moving through the canopy, repositioning on branches and even engaging in social interactions throughout the night, providing direct visual evidence of fragmented sleep in wild primates. Such nocturnal wakefulness and sleep fragmentation are increasingly documented across wild primate taxa—in olive baboons using accelerometery ( *Papio Anubis* ; Loftus et al. 2022), in chimpanzees using both terrestrial camera traps ( *Pan troglodytes* ; Tagg et al. 2018) and infrared camera systems (Hozer et al. 2026), and in Javan slow lorises using actigraphy ( *Nycticebus javanicus* ; Reinhardt et al. 2019)—suggesting that sleep fragmentation may be a widespread feature in wild primates.

This study demonstrates that sleeping sites represent a key, and perhaps overlooked, ecological resource structuring primate community dynamics. By revealing both spatial and temporal segregation in sleeping‐site use, our findings shed light on the mechanisms of resource partitioning, interspecific contest competition and adaptive behavioural strategies operating within this primate community. Given that *C. trapezifolia* follows a supra‐annual fruiting pattern, and that these trees were already intensively selected and contested during our non‐fruiting study period, a compelling perspective for future research will be to examine how competition is reshaped during a fruiting year, when the same trees simultaneously represent a preferred sleeping site and a rich food source. While this study explored sleeping‐site use at the species level, future studies that incorporate reliable individual identification—potentially aided by rapid advances in AI‐assisted classification methods (Gao et al. 2026; Guo et al. 2020; Paulet et al. 2024; Xu et al. 2024)—would be valuable for monitoring group and subgroup dynamics, demographic composition and long‐term patterns of sleeping‐site use by known individuals. Beyond sleeping‐site selection itself, arboreal camera traps proved a promising tool for capturing nocturnal behaviour, documenting sleep fragmentation, and linking sleep ecology and quality to both biotic and abiotic drivers. More broadly, this approach offers a scalable, non‐invasive framework for investigating fine‐scale ecological dynamics in arboreal systems, with wide applications for comparative studies seeking to reconstruct primate behavioural evolution and unravel the complex adaptations of mammals living in the canopy.

## Author Contributions

**Olivier Kaisin:** conceptualization (lead), data curation (equal), formal analysis (equal), investigation (equal), methodology (equal), supervision (equal), visualization (equal), writing – original draft (lead), writing – review and editing (equal). **Mathéo Pochez:** data curation (equal), formal analysis (equal), investigation (equal), visualization (equal), writing – original draft (supporting), writing – review and editing (equal). **Cédric Vermeulen:** conceptualization (equal), funding acquisition (supporting), investigation (equal), methodology (equal), supervision (equal), writing – review and editing (equal). **Laurence Culot:** conceptualization (lead), funding acquisition (lead), methodology (equal), project administration (lead), resources (equal), supervision (equal), writing – review and editing (equal).

## Funding

This work was supported by Fundação de Amparo à Pesquisa do Estado de São Paulo, Young Investigator #2021/06668‐0, Postdoctoral Fellow #2025/06533‐9, CBioClima #2021/10639‐5. Duesberg Fondation, University of Liège. Wallonie‐Bruxelles International. Erasmus+. Conselho Nacional de Desenvolvimento Científico e Tecnológico, 305215/2025‐6.

## Conflicts of Interest

The authors declare no conflicts of interest.

## Supporting information


**Appendix S1:** Botanical survey method.
**Table S1:** Ethogram of the main behaviours used to assess activity budgets in the copaíba trees (*Copaifera trapezifolia*).
**Table S2:** List of the species detected in the monitored copaíbas (*Copaifera trapezifolia*).
**Table S3:** Number of estimated nights spent by each primate species in the 10 monitored copaíbas (*Copaifera trapezifolia*).
**Table S4:** Maximum numbers of individuals, by sex–age categories, recorded in a single detection for each primate species at the 10 monitored copaíbas (*Copaifera trapezifolia*).
**Table S5:** Nights during which multiple monitored copaíbas were used as sleeping sites by different primate species, groups and/or subgroups.
**Table S6:** Model selection results for the effects of abiotic variables on nocturnal presence in copaíba trees (*Copaifera trapezifolia*) for each primate species.
**Table S7:** List of interspecific encounters between primate species in the monitored copaíbas.
**Figure S1:** Density distributions of primate occurrences in each monitored copaíba tree (C1–C10; *Copaifera trapezifolia*). Density of occurrences was calculated per day based on all 15‐min camera trap records and smoothed. Colours represent species: muriquis ( *Brachyteles arachnoides* ; green), howlers ( *Alouatta guariba* ; red) and capuchins (*Sapajus nigritus cucullatus*; blue). Black arrows indicate interspecific interactions between primate species.
**Figure S2:** Diurnal activity budget of primate species in the copaíbas (*Copaifera trapezifolia*) based on camera trap detections. Detections were considered as scan samples of the visible individuals and regrouped into visits. Muriquis ( *Brachyteles arachnoides* ), howlers ( *Alouatta guariba* ) and capuchins (*Sapajus nigritus cucullatus*)
**Figure S3:** Twilight activity budget of primate species in the copaíbas (*Copaifera trapezifolia*) based on observed behaviour in camera trap detections only. Detections were considered as scan samples of the visible individuals and regrouped into visits. Muriquis ( *Brachyteles arachnoides* ), howlers ( *Alouatta guariba* ) and capuchins (*Sapajus nigritus cucullatus*).
**Figure S4:** Core nighttime activity budget of primate species in the copaíbas (Copaifera trapezifolia) based on observed behaviour in camera trap detections only. Detections were considered as scan samples of the visible individuals and regrouped into visits. Muriquis ( *Brachyteles arachnoides* ), howlers ( *Alouatta guariba* ) and capuchins (*Sapajus nigritus cucullatus*).

**Video S1:** Examples of daytime activity by southern muriquis (*Brachyteles arachnoides*), brown howler monkeys (*Alouatta guariba*), and black‐tufted capuchins (*Sapajus nigritus cucullatus*) in *Copaifera trapezifolia* trees, recorded by arboreal camera traps.
**Video S2:** Examples of nocturnal activity by southern muriquis (*Brachyteles arachnoides*), brown howler monkeys (*Alouatta guariba*), and black‐tufted capuchins (*Sapajus nigritus cucullatus*) in *Copaifera trapezifolia* sleeping sites, recorded by arboreal camera traps.
**Video S3:** Examples of interspecific encounters involving southern muriquis (*Brachyteles arachnoides*), brown howler monkeys (*Alouatta guariba*), and black‐tufted capuchins (*Sapajus nigritus cucullatus*) in *Copaifera trapezifolia* trees, recorded by arboreal camera traps.
**Video S4:** Examples of fur‐rubbing by southern muriquis (*Brachyteles arachnoides*), brown howler monkeys (*Alouatta guariba*), and black‐tufted capuchins (*Sapajus nigritus cucullatus*) on *Copaifera trapezifolia* trees, recorded by arboreal camera traps.

## Data Availability

The data that support the findings of this study are available in the Dryad Digital Repository: https://doi.org/10.5061/dryad.j3tx95xws.
