# Supplementary material for: Lights Out in the Canopy: Arboreal Camera Traps Offer New Insights Into Sleeping‐Site Partitioning and Sleep Ecology in Primate Communities
Source: Ecol Evol. 2026 Oct 28;16(11):e74525. doi: 10.1002/ece3.74525 (PMC13714698; doi:10.1002/ece3.74525)
Supplement: Supplementary file 1 [file ECE3-16-e74525-s002.docx]

**SUPPLEMENTARY INFORMATION**

Lights out in the canopy: arboreal camera traps offer new insights into sleeping-site partitioning and sleep ecology in primate communities

KAISIN Olivier, POCHEZ Mathéo, VERMEULEN Cédric, CULOT Laurence

**Appendix S1.** Botanical survey method

To estimate the abundance of *C. trapezifolia* and compare the diameter at breast height (DBH) and height of the monitored trees with those of large trees generally available in the study area, we conducted a botanical survey of 100 plots measuring 10 × 10 m. The plots were established in a grid at 500-m intervals across 20 locations, corresponding to the locations where acoustic recording units (ARUs) were deployed by the Laboratory of Primatology (UNESP, Rio Claro). The 20 locations were arranged in four rows of five locations. At each ARU location, five 10 × 10 m plots were established: one at the centre and one in each of the four cardinal directions, with adjacent plots spaced 50 m apart. Within each plot, all trees with a DBH > 5 cm were measured and identified, following the methodology used by Souza et al. (2018)^[1]^. Tree height was measured using a rangefinder. The 100 plots covered a total area of 1 ha, corresponding to approximately 0.71% of the area encompassed by the monitored copaíba trees (141.23 ha).

#### **Table S1**. Ethogram of the main behaviours used to assess activity budgets in the copaíba trees (Copaifera trapezifolia).

| Main behaviour | Description |
| --- | --- |
| *Foraging* | Searching for, handling, or consuming food items. |
| *Inactive* | Stationery and alert (e.g. standing or sitting while scanning the surroundings), without feeding or social interaction. |
| *Playing* | Non-aggressive social interactions, including climbing on conspecifics, chasing, or play biting. |
| *Moving* | Locomotion within the camera’s field of view while remaining in the same tree. |
| *Traveling* | Directed locomotion exiting the camera’s field of view. |
| *Resting* | Sitting or lying inactive, including sleeping or inactive states without vigilance. |
| *Sleeping* | Directed locomotion exiting the camera’s field of view. |

***Table* S2.** List of the species detected in the monitored copaíbas (*Copaifera trapezifolia*)*.*

| Scientific name | Common name | Number of detections |
| --- | --- | --- |
| *Brachyteles arachnoides* | Southern Muriqui | 2850 |
| *Alouatta guariba* | Brown Howler | 737 |
| *Sapajus nigritus cucullatus* | Southern black-tufted Capuchin | 608 |
| Family: Didelphidae | Opossum | 23 |
| *Nasua nasua* | South American Coati | 19 |
| *Coendou spinosus* | Brazilian Porcupine | 13 |
| *Eira barbara* | Tayra | 5 |
| *Pipile jacutinga* | Black-fronted Piping-guan | 5 |
| *Penelope obscura* | Dusky-legged Guan | 4 |
| *Didelphis aurita* | Brazilian Common Opossum | 4 |
| Family: Dendrocolaptidae | Woodcreeper | 4 |
| Order: Rodentia | Rodent | 3 |
| *Ramphastos dicolorus* | Red-breasted Toucan | 3 |
| *Celeus flavescens* | Blond-crested Woodpecker | 2 |
| *Tamandua tetradactyla* | Southern Tamandua | 2 |
| *Turdus flavipes* | Yellow-legged Thrush | 1 |
| *Pyroderus scutatus* | Red-ruffed Fruitcrow | 1 |
| *Trogon surucua* | Southern Surucua Trogon | 1 |
| *Spizaetus tyrannus* | Black Hawk-eagle | 1 |
| *Pyrrhura frontalis* | Maroon-bellied Parakeet | 1 |
| *Thraupis cyanoptera* | Azure-shouldered tanager | 1 |

***Table* S3*.*** Number of estimated nights spent by each primate species in the 10 monitored copaíbas (*Copaifera trapezifolia*).

| Species | C1 | C2 | C3 | C4 | C5 | C6 | C7 | C8 | C9 | C10 | Total |
| --- | --- | --- | --- | --- | --- | --- | --- | --- | --- | --- | --- |
| *Brachyteles arachnoides* | 33 | 4 | 45 | 6 | 2 | 1 | 1 | 0 | 1 | 1 | 94 |
| *Alouatta guariba* | 3 | 4 | 5 | 1 | 1 | 5 | 1 | 1 | 1 | 0 | 22 |
| *Sapajus nigritus cucullatus* | 3 | 13 | 0 | 0 | 1 | 4 | 16 | 0 | 4 | 1 | 42 |
| Total | 39 | 21 | 50 | 7 | 4 | 10 | 18 | 1 | 6 | 2 | 158 |

***Table* S4*.*** Maximum numbers of individuals, by sex -age categories, recorded in a single detection for each primate species at the 10 monitored copaíbas (*Copaifera trapezifolia*).

| **Tree ID** | **Species** | **# individuals** | **Adult male** | **Adult female** | **Subadult** | **Juvenile** | **Infant** | **Total^2^** |
| --- | --- | --- | --- | --- | --- | --- | --- | --- |
| **C1** | Muriquis | 8 | 3 | 3 | 3 | 1 | 4 | 14 |
|  | Howlers | 5 | 1 | 1 | 0 | 0 | 1 | 5 |
|  | Capuchins | 2 | 0 | 1 | 1 | 0 | 1 | 2 |
| **C2** | Muriquis | 5 | 3 | 2 | 1 | 1 | 2 | 9 |
|  | Howlers | 6 | 2 | 2 | 1 | 1 | 1 | 7 |
|  | Capuchins | 9 | 2 | 3 | 2 | 1 | 5 | 13 |
| **C3** | Muriquis | 7 | 4 | 3 | 2 | 1 | 3 | 13 |
|  | Howlers | 6 | 2 | 1 | 0 | 0 | 3 | 6 |
|  | Capuchins | 1 | 1 | 0 | 0 | 0 | 1 | 2 |
| **C4** | Muriquis | 6 | 3 | 4 | 1 | 1 | 2 | 11 |
|  | Howlers | 5 | 1 | 1 | 0 | 1 | 1 | 5 |
| **C5** | Muriquis | 9 | 4 | 3 | 2 | 1 | 3 | 13 |
|  | Howlers | 4 | 1 | 1 | 0 | 1 | 1 | 4 |
|  | Capuchins | 5 | 2 | 2 | 1 | 1 | 3 | 9 |
| **C6** | Muriquis | 7 | 4 | 2 | 0 | 0 | 4 | 10 |
|  | Howlers | 4 | 1 | 1 | 0 | 0 | 1 | 4 |
|  | Capuchins | 3 | 1 | 1 | 1 | 0 | 2 | 5 |
| **C7** | Muriquis | 8 | 2 | 3 | 1 | 1 | 3 | 10 |
|  | Howlers | 4 | 1 | 1 | 0 | 0 | 1 | 4 |
|  | Capuchins | 8 | 2 | 2 | 3 | 1 | 4 | 12 |
| **C8** | Muriquis | 6 | 3 | 2 | 2 | 1 | 2 | 10 |
|  | Howlers | 4 | 2 | 1 | 0 | 1 | 1 | 5 |
|  | Capuchins | 3 | 1 | 1 | 2 | 1 | 2 | 7 |
| **C9** | Muriquis | 7 | 3 | 5 | 2 | 1 | 2 | 13 |
|  | Howlers | 5 | 1 | 1 | 1 | 0 | 1 | 5 |
|  | Capuchins | 7 | 1 | 1 | 1 | 1 | 5 | 9 |
| **C10** | Muriquis | 3 | 1 | 1 | 0 | 1 | 0 | 3 |
|  | Howlers | 2 | 1 | 0 | 0 | 0 | 0 | 2 |
|  | Capuchins | 2 | 0 | 0 | 0 | 0 | 1 | 2 |

*Note*: Muriquis (*Brachyteles arachnoides*), howlers (*Alouatta guariba*), and capuchins (*Sapajus nigritus cucullatus*).^1^Maximum number of individuals observed in a single detection. ^2^Sum of the maximum numbers recorded independently for each sex–age category at each tree. These maxima were not necessarily obtained from the same videos and may not refer to the same individuals or social groups.

***Figure* S1.** Density distributions of primate occurrences in each monitored copaíba tree (C1–C10; *Copaifera trapezifolia*). Density of occurrences was calculated per day based on all 15-minute camera trap records and smoothed. Colours represent species: muriquis (*Brachyteles arachnoides*; green), howlers (*Alouatta guariba*; red), and capuchins (*Sapajus nigritus cucullatus*; blue). Black arrows indicate interspecific interactions between primate species.

***Table* S5.** Nights during which multiple monitored copaíbas were used as sleeping sites by different primate species, groups and/or subgroups.

| **Date** | **Number of occupied trees** | **Tree IDs** | **Primate species** |
| --- | --- | --- | --- |
| 13/12/2024 | 2 | C1, C7 | Muriquis, Capuchins |
| 15/12/2024 | 2 | C1, C3 | Muriquis, Muriquis |
| 16/12/2024 | 2 | C1, C2 | Muriquis, Capuchins |
| 24/12/2024 | 2 | C1, C6 | Muriquis, Capuchins |
| 27/12/2024 | 2 | C1, C2 | Muriquis, Capuchins |
| 03/01/2025 | 2 | C3, C5 | Muriquis, Muriquis |
| 06/01/2025 | 2 | C3, C7 | Muriquis, Capuchins |
| 07/01/2025 | 3 | C1, C3, C7 | Muriquis, Muriquis, Capuchins |
| 14/01/2025 | 3 | C1, C3, C7 | Muriquis, Muriquis, Capuchins |
| 16/01/2025 | 3 | C3, C9, C7 | Muriquis, Muriquis, Capuchins |
| 17/01/2025 | 2 | C3, C6 | Muriquis, Muriquis |
| 15/02/2025 | 2 | C1, C3 | Muriquis, Muriquis |
| 25/02/2025 | 2 | C3, C7 | Muriquis, Capuchins |
| 26/02/2025 | 2 | C1, C3 | Muriquis, Muriquis |
| 28/02/2025 | 2 | C3, C2 | Muriquis, Capuchins |
| 09/03/2025 | 3 | C3, C1, C7 | Muriquis, Capuchins, Capuchins |
| 10/03/2025 | 2 | C1, C3 | Muriquis, Muriquis |
| 13/03/2025 | 2 | C3, C6 | Muriquis, Capuchins |
| 14/03/2025 | 2 | C1, C2 | Muriquis, Capuchins |
| 16/03/2025 | 2 | C3, C4 | Muriquis, Muriquis |
| 17/03/2025 | 2 | C1, C3 | Muriquis, Muriquis |
| 19/03/2025 | 2 | C1, C7 | Muriquis, Capuchins |
| 23/03/2025 | 3 | C1, C3, C6 | Muriquis, Muriquis, Capuchins |
| 24/03/2025 | 2 | C1, C3 | Muriquis, Muriquis |
| 27/03/2025 | 2 | C1, C3 | Muriquis, Muriquis |
| 28/03/2025 | 2 | C10, C3 | Muriquis, Muriquis |
| 02/04/2025 | 2 | C1, C2 | Capuchins, Capuchins |
| 09/04/2025 | 2 | C3, C1 | Howlers, Muriquis |
| 10/04/2025 | 2 | C2, C9 | Capuchins, Capuchins |
| 11/04/2025 | 3 | C2, C2, C9 | Muriquis, Capuchins, Capuchins |
| 12/04/2025 | 2 | C3, C3 | Howlers, Muriquis |
| 14/04/2025 | 2 | C3, C4 | Muriquis, Muriquis |
| 05/05/2025 | 2 | C2, C2 | Howlers, Capuchins |
| 21/05/2025 | 2 | C1, C3 | Howlers, Howlers |
| 05/06/2025 | 2 | C9, C1 | Howlers, Muriquis |
| 06/06/2025 | 3 | C1, C10, C2 | Muriquis, Capuchins, Capuchins |

*Note*: Muriquis (*Brachyteles arachnoides*), howlers (*Alouatta guariba*), and capuchins (*Sapajus nigritus cucullatus*).

***Table* S6.** Model selection results for the effects of abiotic variables on nocturnal presence in copaíba trees (*Copaifera trapezifolia*) for each primate species

| Species | Model | AIC | ΔAIC | LRT *p*-value (vs. null) |
| --- | --- | --- | --- | --- |
| Muriquis | *Minimal temperature + wind speed + moon phase* | *219.67* | *0.00* | *<0.001* |
|  | Global model | 221.66 | 1.99 |  |
|  | Null model | 237.36 | 17.70 |  |
| Howlers | Null model | 106.88 | 0.00 | — |
|  | Global model | 113.36 | 6.48 |  |
| Capuchins | *Minimal temperature + moon phase* | *151.61* | *0.00* | *0.012* |
|  | Global model | 154.93 | 3.32 |  |
|  | Null model | 156.52 | 4.91 |  |

*Note*: Models were selected using bidirectional stepwise selection based on Akaike's information criterion (AIC). ΔAIC represents the difference in AIC relative to the selected model. The LRT p-value corresponds to a likelihood ratio test comparing the selected model with the null model. The best-supported model is shown in italics.

***Figure* S2.** Diurnal activity budget of primate species in the copaíbas (*Copaifera trapezifolia*) based on camera trap detections. Detections were considered as scan samples of the visible individuals and regrouped into visits. Muriquis (*Brachyteles arachnoides*), howlers (*Alouatta guariba*), and capuchins (*Sapajus nigritus cucullatus*).

***Figure* S3.** Twilight activity budget of primate species in the copaíbas (*Copaifera trapezifolia*) based on observed behaviour in camera trap detections only. Detections were considered as scan samples of the visible individuals and regrouped into visits. Muriquis (*Brachyteles arachnoides*), howlers (*Alouatta guariba*), and capuchins (*Sapajus nigritus cucullatus*).

***Figure* S4.** Core nighttime activity budget of primate species in the copaíbas (*Copaifera trapezifolia*) based on observed behaviour in camera trap detections only. Detections were considered as scan samples of the visible individuals and regrouped into visits. Muriquis (*Brachyteles arachnoides*), howlers (*Alouatta guariba*), and capuchins (*Sapajus nigritus cucullatus*).

***Table* S7.** List of interspecific encounters between primate species in the monitored copaíbas

| **TreeID** | **Date** | **Species pair** | **Start** | **End** | **Duration** | **Sleeping night**^1^ | **Species first present** | **Species that remained**^2^ |
| --- | --- | --- | --- | --- | --- | --- | --- | --- |
| C1 | 05/06/2025 | Howler - Muriqui | 17:24 | 17:27 | 3 | Yes | Muriqui | Muriqui |
| C2 | 11/04/2025 | Muriqui - Capuchin | 18:08 | 18:30 | 21.4 | Yes | Muriqui | Capuchin |
| C2 | 05/05/2025 | Howler - Capuchin | 17:53 | 17:53 | 0.5 | Yes | Howler | Howler |
| C2 | 16/05/2025 | Howler - Muriqui | 11:32 | 11:35 | 2.93 | No | Howler | Muriqui |
| C3 | 12/12/2024 | Howler - Muriqui | 15:52 | 16:10 | 18.7 | No | Howler | Muriqui |
| C3 | 29/12/2024 | Howler - Muriqui | 16:50 | 16:57 | 6.88 | Yes | Howler | Muriqui |
| C3 | 29/01/2025 | Howler - Muriqui | 11:05 | 11:09 | 3.92 | No | Howler | Muriqui |
| C3 | 05/03/2025 | Howler - Muriqui | 09:49 | 09:56 | 7.03 | No | Howler | Muriqui |
| C3 | 13/04/2025 | Howler - Muriqui | 07:31 | 07:45 | 14.5 | Yes | Howler | Muriqui |
| C3 | 22/05/2025 | Howler - Muriqui | 10:12 | 10:12 | 0.5 | Yes | Howler | Muriqui |
| C6 | 05/02/2025 | Howler - Muriqui | 07:41 | 07:45 | 3.43 | Yes | Howler | Muriqui |
| C8 | 20/04/2025 | Howler - Muriqui | 10:30 | 11:00 | 29.54 | No | Howler | Muriqui |
| C8 | 30/04/2025 | Muriqui - Capuchin | 11:57 | 12:09 | 11.9 | No | Muriqui | / |

*Note*: Muriquis (*Brachyteles arachnoides*), howlers (*Alouatta guariba*), and capuchins (*Sapajus nigritus cucullatus*). ^1^Sleeping night indicates whether at least one species involved in the encounter used the copaíba as a sleeping site that night. ^2^Species that remained refers to the species still present in the copaíba following the encounter. (/) indicates that neither species remained.

1. Souza, A. C. O. de, Benacci, L., & Joly, C. A. (2018). Floristic and structure of the arboreal community of an Ombrophilous Dense Forest at 800 m above sea level, in Ubatuba/SP, Brazil. *Biota Neotropica*, 18(4). https://doi.org/10.1590/1676-0611-bn-2018-0590
